# Diagnostic Usefulness of Liquid Culture Medium for Mycobacterium avium-intracellulare Complex Lung Disease: A Single-Centre, Retrospective Study

**DOI:** 10.7759/cureus.61542

**Published:** 2024-06-02

**Authors:** Hiroshi Kobe, Akihiro Ito, Yosuke Nakanishi, Yui Miyazaki, Hiroshi Takahashi, Yushi Toyota, Akihiko Amano, Kyoko Matsui, Tadashi Ishida

**Affiliations:** 1 Respiratory Medicine, Kurashiki Central Hospital, Kurashiki, JPN

**Keywords:** radiological pattern, smear-positive, ogawa culture, mycobacterium avium-intracellulare complex, liquid culture

## Abstract

Background

The diagnosis of *Mycobacterium avium-intracellulare* complex lung disease (MAC-LD) requires two or more positive sputum cultures. Few reports have examined the usefulness of adding liquid culture to conventional solid culture for diagnosing MAC-LD.

Methods

A retrospective, cohort study of patients examined at Kurashiki Central Hospital in Japan with a confirmed diagnosis of MAC-LD between January 1, 2002, and June 20, 2021, was conducted. The primary endpoint was the culture positivity rate, which was compared between the liquid and Ogawa culture media in patients who underwent sputum culture using both methods. Secondary endpoints were the culture positivity rate in smear-positive specimens and the positivity rate by radiological type.

Results

The study, which involved 351 patients and 702 specimens, showed a higher positivity rate for liquid culture (n=690, 98.3%) than Ogawa culture (n=315, 44.9%). Overall, 265 patients (75.5%) would have had delayed MAC-LD diagnosis without liquid medium being used. Of the 95 smear-positive specimens, 71 (74.7%) were positive on both cultures, whereas 24 (25.3%) were positive only on liquid culture. The positivity rate of Ogawa culture varied by radiological type.

Conclusions

Liquid culture is more valuable for the early diagnosis of MAC-LD than Ogawa culture.

## Introduction

Nontuberculous mycobacteria (NTM) species are ubiquitous in the environment. NTM species include over 190 species and subspecies, some of which can produce disease in humans of all ages and can affect both pulmonary and extrapulmonary sites [1]. Population-based data have documented a worldwide increase in the prevalence of human NTM infection since 2000 [2]. In 2014, the incidence rate of NTM infection was estimated to be 14.7 cases per 100,000 person-years, which is about 2.6 times the incidence rate reported in 2007 in Japan [3]. Of the NTM species, *Mycobacterium avium-intracellulare* complex (MAC) was the most commonly isolated species (93.3%) [4]. MAC lung disease (MAC-LD) often progresses slowly, and it is important to diagnose properly and follow it over time. An official clinical practice guideline showed that NTM can be isolated from respiratory specimens due to environmental contamination and some patients who have NTM isolated from their respiratory tract do not show evidence of progressive disease [1]. Therefore, more than one positive sputum culture of the same NTM species is recommended for diagnostic purposes [1]. When MAC-LD is strongly suspected on chest imaging, but not diagnosed on sputum examination, bronchoscopy should be considered. Several studies have reported that bronchoscopy has a diagnostic yield of 52.8% and is more sensitive than sputum culture [5,6]. Patients who were diagnosed by sputum specimens tended to have more frequent and severe cough, sputum, and postnasal drip than those diagnosed by bronchoscopic specimens. There is some reluctance to perform highly invasive bronchoscopy in patients with milder symptoms. Therefore, higher diagnostic sensitivity of sputum examination is needed. Regarding sputum culture media, liquid and Ogawa media (solid media) are used to cultivate mycobacteria. In the diagnosis of tuberculosis, liquid medium gives higher yield and faster results than solid medium [7]. However, few reports have researched the usefulness of liquid media in the diagnosis of MAC-LD. The aim of this study was to investigate the usefulness of liquid medium compared with Ogawa medium for diagnosing MAC-LD.

## Materials and methods

Study design and patients

This was a single-centre, retrospective, cohort study. A chart review of patients whose sputum specimens showed MAC at least twice from January 1, 2002, to June 30, 2021, was conducted. Only specimens collected from treatment-naïve MAC cases were included. Exclusion criteria were sputum that had not been tested with both media and sputum collected from patients without pulmonary lesions. This study was approved by the Ethics Committee for Clinical Studies of Kurashiki Central Hospital (approval number: 4059), which waived the requirement for informed consent due to the retrospective nature of the study. Patients' characteristics including age, sex, body mass index, performance status, comorbidities, immunosuppressant therapy, radiological findings, and bacterial species were investigated. MAC-LD was diagnosed on the basis of the American Thoracic Society (ATS)/Infectious Diseases Society of America (IDSA) 2007 criteria [1].

Cultivation media

**Figure 1:**
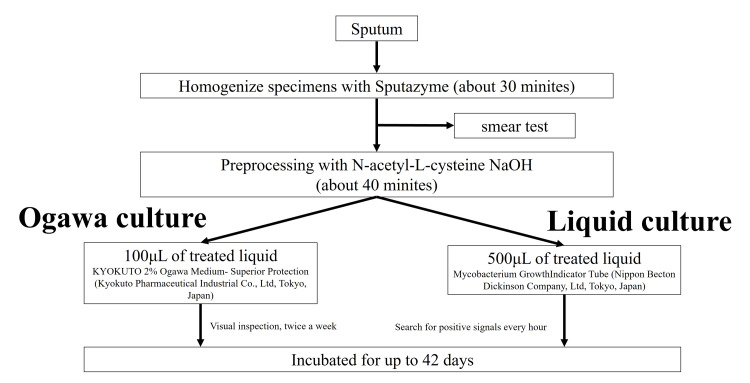
Sputum specimen processing policy at Kurashiki Central Hospital.

Until January 21, 2012, KYOKUTO 3% Ogawa Medium-Superior Protection (Kyokuto Pharmaceutical Industrial Co., Ltd., Tokyo, Japan) had been used as the solid cultivation medium. Sputum was treated with 8% sodium hydroxide, and specimens other than sputum were treated with 3% sodium hydroxide; 0.1 mL of specimen solution was inoculated into two bottles of 3% Ogawa medium and incubated for eight weeks. Since January 31, 2012, YOKUTO 2% Ogawa Medium-Superior Protection was used as the solid cultivation medium. Mycobacterium Growth Indicator Tube (MGIT) (Nippon Becton Dickinson Company, Ltd., Tokyo, Japan) was used as the liquid cultivation medium. Samples were treated with semi-alkaline-protease and N-acetyl-L-cysteine-NaOH pre-treatment with Sputazyme (Kyokuto Pharmaceutical); 0.1 mL of specimen solution was inoculated into a bottle of 2% Ogawa medium, and 0.5 mL was inoculated into a bottle of liquid medium, and both media were incubated for 6-8 weeks (Figure 1).

A positive sputum specimen on liquid culture (LC) is denoted by Lp and a negative specimen by Ln, and similarly, a positive specimen on Ogawa culture (OC) is denoted by Op and a negative specimen by On. The combination of two sputum test results for one patient is denoted LpOp-LpOp (positive twice for LC and OC), LpOp-LpOn (positive once for both LC and OC and positive once only for LC), LpOp- LnOp (positive once for both LC and OC and once only for OC), LpOn-LpOn (positive only for LC twice), LnOp-LnOp (positive only for OC twice), and LpOn-LnOp (positive once only for LC and once only for OC).

Criteria for the selection of culture media

Sputum was cultured by either or both MGIT liquid medium and Ogawa medium. Which medium was used depended on the policy of our hospital's bacteriology laboratory. Until January 31, 2012, the specimens were cultured only on Ogawa medium. From February 1, 2012, MGIT liquid medium was introduced, and since then, all cases have been cultured on liquid medium. When the following criteria were met, Ogawa medium was also used for sputum culture submitted at the outpatient clinic in our hospital and airway specimens collected by bronchoscopy, whereas only liquid medium was used for sputum from hospitalized patients.

Radiological patterns

**Figure 2:**
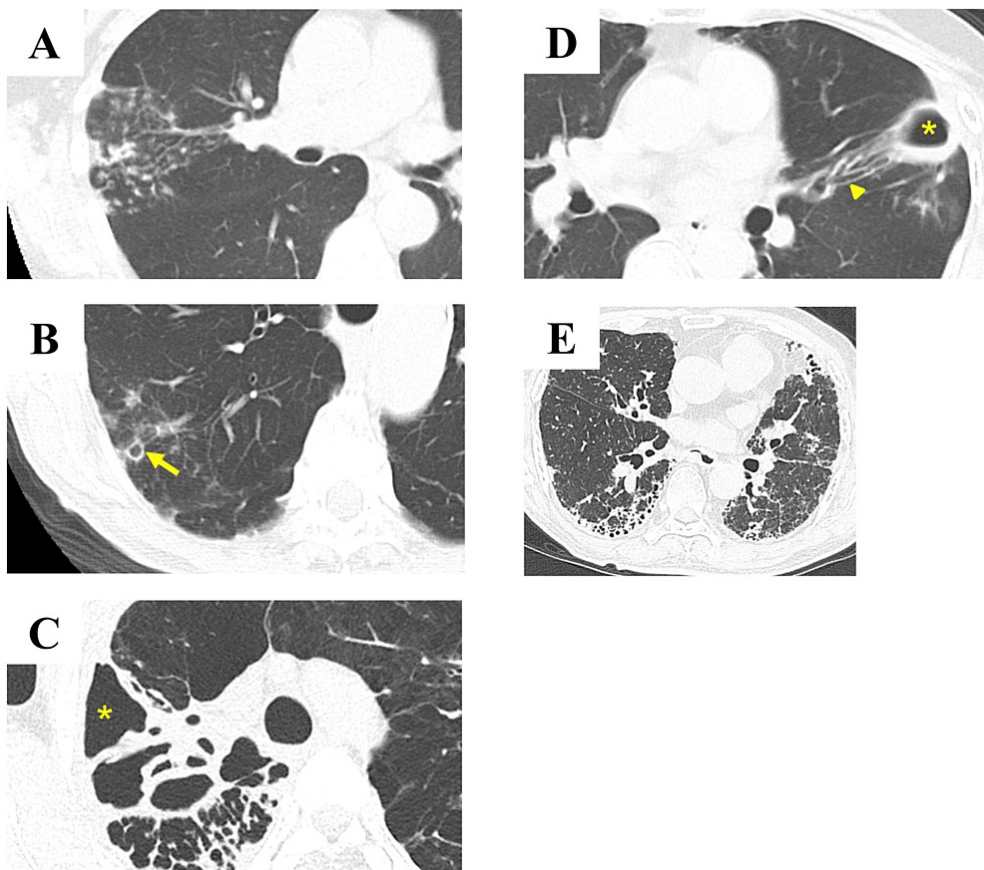
Examples of chest computed tomography findings in each radiological pattern. (A) Non-cavitary NB type, nodules and bronchiectasis without cavity. (B) Cavitary NB type, nodules and bronchiectasis type with one or more cavities (yellow arrow). (C) FC type, fibrocavitary (asterisk). (D) NB+FC type, type with characteristics of both NB and FC types, fibrocavitary (asterisk) and bronchiectasis (arrowhead). (E) Unclassifiable, radiological patterns that cannot be classified as any of the above. FC: fibrocavitary; NB: nodular bronchiectatic

Chest computed tomography (CT) images were classified into five categories (Figure 2): (A) non-cavitary NB type, with nodules and bronchiectasis without a cavity; (B) cavitary NB type, with nodules and bronchiectasis type with one or more cavities; (C) FC type, fibrocavitary; (D) NB+FC type, type with characteristics of both NB and FC type; and (E) unclassifiable, types that could not be classified into the above four categories. Chest CT features were confirmed by three respiratory physicians (HK, AI, and YN).

Outcomes

The primary aim was to compare the primary endpoint, the culture positivity rate, between the two culture media in patients who underwent sputum culture using both media twice. The secondary endpoints included the positivity rates in smear-positive specimens and by radiological patterns.

Statistical analyses

Data are presented as means or numbers (percentages). Categorical variables were analyzed using Fisher's exact test, and continuous variables were analyzed using the non-parametric Mann-Whitney U test. All p-values were two-sided, and p-values of 0.05 or less were considered significant. All statistical analyses were performed with EZR [8], which is a graphical user interface for R (The R Foundation for Statistical Computing, Vienna, Austria, Version 4.0.3). More precisely, it is a modified version of R commander designed to add statistical functions frequently used in biostatistics.

## Results

Patients

**Figure 3:**
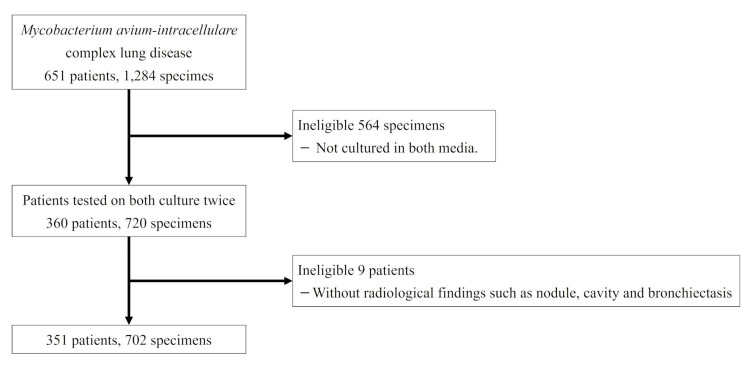
Study flowchart.

A total of 351 patients (702 specimens) were analyzed in this study (Figure 3).

The patients' characteristics are shown in Table 1. The mean age of the patients was 70.9 years, and 243 (69.2%) were female. Underlying respiratory diseases included bronchial asthma (n=9, 2.6%), chronic obstructive pulmonary disease (n=29, 8.3%), interstitial pneumonia (n=16, 4.6%), and old tuberculosis (n=20, 5.7%). Radiological features included non-cavitary NB type (n=273, 77.8%), cavitary NB (n=41, 11.7%), FC (n=13, 3.7%), NB+FC (n=7, 2.0%), and unclassifiable (n=17, 4.8%). NTM species included *M. avium* (n=37, 10.5%), *M. intracellulare* (n=72, 20.5%), and MAC (n=242, 69.0%).

**Table 1:** Characteristics of patients with Mycobacterium avium-intracellulare complex lung disease. *Only active cases currently under treatment or on treatment-free follow-up were included; recurrence-free more than one year after surgery not included. BMI: body mass index; ECOG: European Cooperative Oncology Group; FC: fibrocavitary; MAC: *Mycobacterium avium-intracellulare* complex; NB: nodular bronchiectatic; NTM: nontuberculous mycobacteria

-	-	Patients (n=351)
Age, y (mean, SD)	-	70.9±9.0
Sex, female, n (%)	-	243 (69.2%)
BMI, kg/m^2^ (mean, SD)	-	19.7±3.3
Smoking	Never	240 (68.4%)
	Current or former	90 (25.6%)
	Not available	21 (6.0%)
ECOG performance status	0-1	288 (82.0%)
	2-4	27 (7.7%)
	Not available	36 (10.3%)
Comorbid disease, n (%)	Bronchial asthma	9 (2.6%)
	Chronic obstructive pulmonary disease	29 (8.3%)
	Interstitial pneumonia	16 (4.6%)
	Old tuberculosis	20 (5.7%)
	Chronic heart disease	11 (3.1%)
	Diabetes mellitus	35 (10.0%)
	Chronic kidney disease	11 (3.1%)
	Gastroesophageal reflux disease	29 (8.3%)
	Liver disorder	9 (2.6%)
	Malignancy*	57 (16.2%)
	Rheumatoid arthritis	17 (4.8%)
	Other connective tissue disease	3 (0.9%)
Immunosuppressant therapy, n (%)	Corticosteroid usage	13 (3.7%)
	Other immunosuppressant drug usage	13 (3.7%)
Radiological pattern, n (%)	Non-cavitary NB	273 (77.8%)
	Cavitary NB	41 (11.7%)
	FC	13 (3.7%)
	NB+FC	7 (2.0%)
	Unclassifiable	17 (4.8%)
NTM species	M. avium	37 (10.5%)
	M. intracellulare	72 (20.5%)
	MAC	242 (69.0%)

Specimen culture results

**Figure 4:**
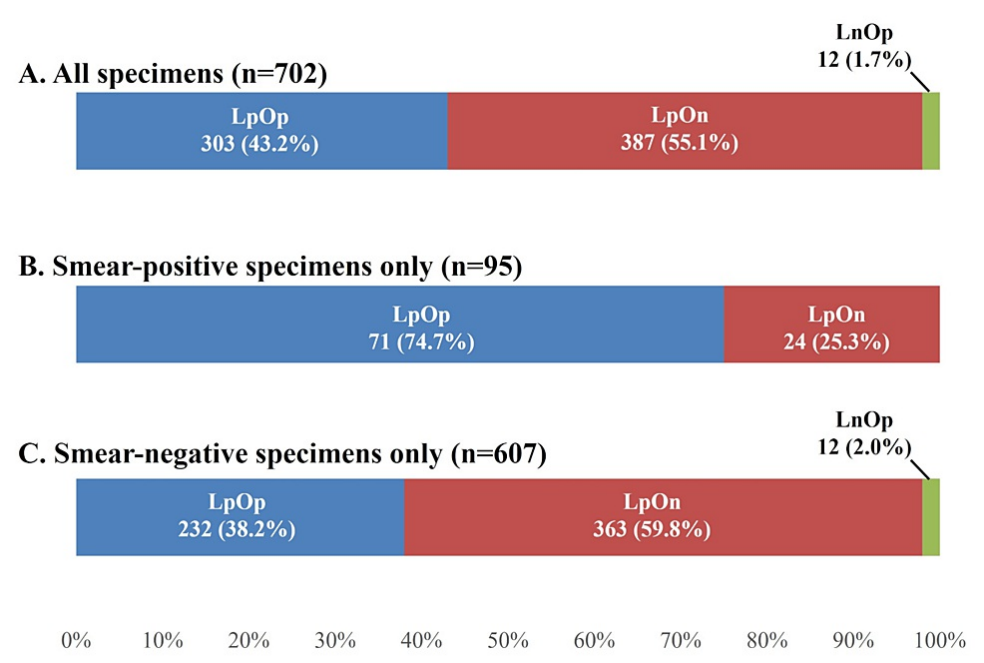
Sputum culture results in liquid and Ogawa culture. (A) Sputum culture results in liquid and Ogawa culture in all specimens. (B) Sputum culture results in liquid and Ogawa culture in smear-positive specimens only. (C) Sputum culture results in liquid and Ogawa culture in smear-negative specimens only. "Lp" indicates a positive result on liquid culture, whereas "Ln" indicates a negative result. Similarly, "Op" indicates a positive result on Ogawa culture, whereas "On" indicates a negative result.

Figure 4 shows a breakdown of the results for the 702 specimens, which included LpOn (n=387, 55.1%), LpOp (n=303, 43.2%), and LnOp (n=12, 1.7%). Of the 95 smear-positive specimens, 71 (74.7%) were LpOp, whereas 24 (25.3%) were LpOn. Of the 607 smear-negative specimens, 232 (38.2%) were LpOp, whereas 363 (59.8%) were LpOn.

**Figure 5:**
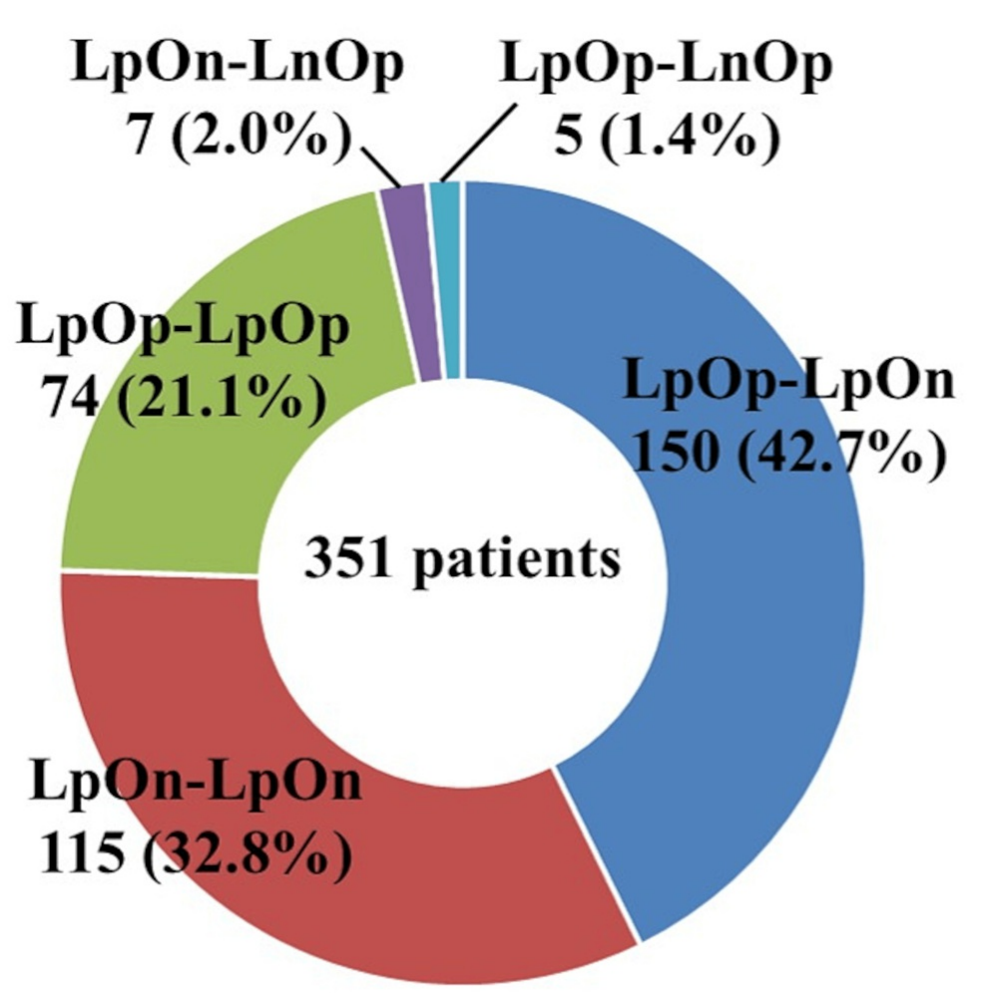
Sputum culture combination for each patient. "Lp" indicates a positive result on liquid culture, whereas "Ln" indicates a negative result. Similarly, "Op" indicates a positive result on Ogawa culture, whereas "On" indicates a negative result. LpOp-LpOp: positive on liquid culture and Ogawa culture twice; LpOn-LpOn: positive on liquid culture twice, but not Ogawa culture; LpOp-LpOn: positive once on both liquid culture and Ogawa culture and once only on liquid culture; LpOp-LnOp: positive once on both liquid culture and Ogawa culture and once only on Ogawa culture; LpOn-LnOp: positive once on liquid culture and once on Ogawa culture

The breakdown of sputum culture results for the 351 patients included LpOp-LnOp (n=5, 1.4%), LpOn-LnOp (n=7, 2.0%), LpOp-LpOp (n=74, 21.1%), LpOn-LpOn (n=115, 32.8%), and LpOp-LpOn (n=150, 42.7%) (Figure 5). Two-hundred and sixty-five (75.5%) patients could only be diagnosed with LC, and using only OC would have delayed diagnosis.

Culture results by radiological pattern

**Figure 6:**
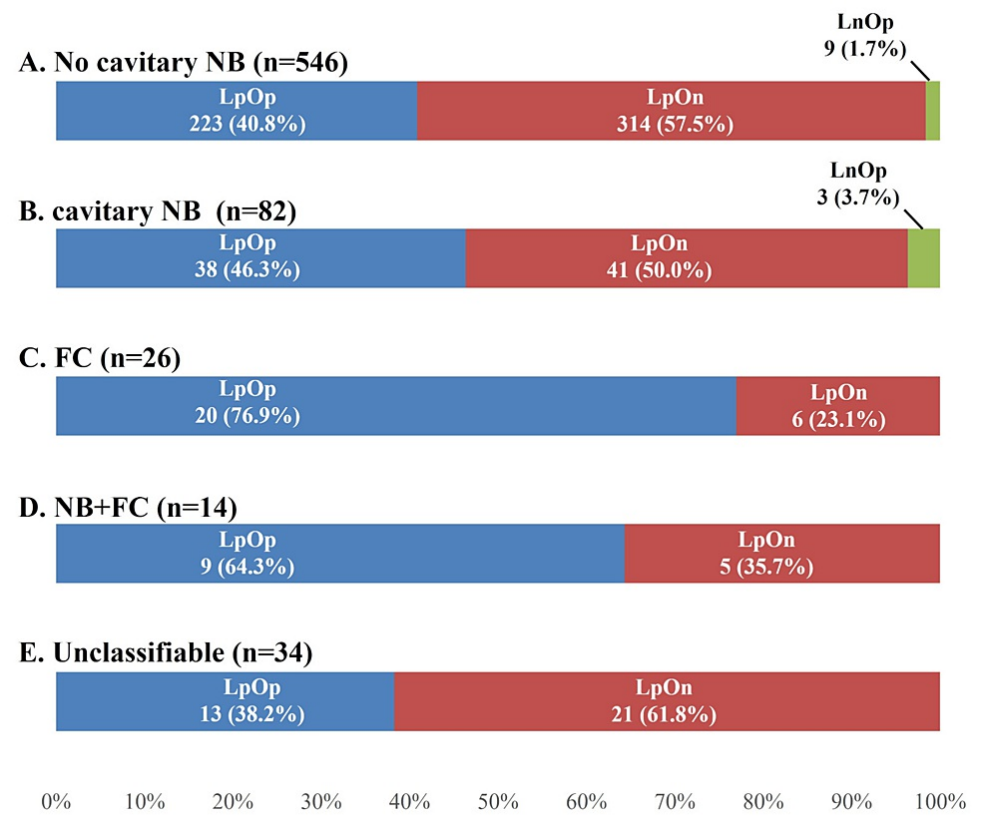
Sputum culture for each radiological pattern. (A) Sputum specimens obtained by patients with no cavitary NB, (B) cavitary NB, (C) FC, (D) NB+FC, and (E) unclassifiable. "Lp" indicates a positive result on liquid culture, whereas "Ln" indicates a negative result. Similarly, "Op" indicates a positive result on Ogawa culture, whereas "On" indicates a negative result. LpOp-LpOp: positive on liquid culture and Ogawa culture twice; LpOn-LpOn: positive on liquid culture twice, but not Ogawa culture; LpOp-LpOn: positive once on both liquid culture and Ogawa culture and once only on liquid culture; LpOp-LnOp: positive once on both liquid culture and Ogawa culture and once only on Ogawa culture; LpOn-LnOp: positive once on liquid culture and once on Ogawa culture; FC: fibrocavitary; NB: nodular bronchiectatic

The breakdown of culture results for each radiological pattern is also presented (Figure 6). The highest positivity rate for OC was observed with FC (n=20, 76.9%), followed by NB+FC (n=9, 64.3%), cavitary NB (n=41, 50.0%), non-cavitary NB (n=232, 42.5%), and unclassifiable cases (n=13, 38.2%).

**Figure 7:**
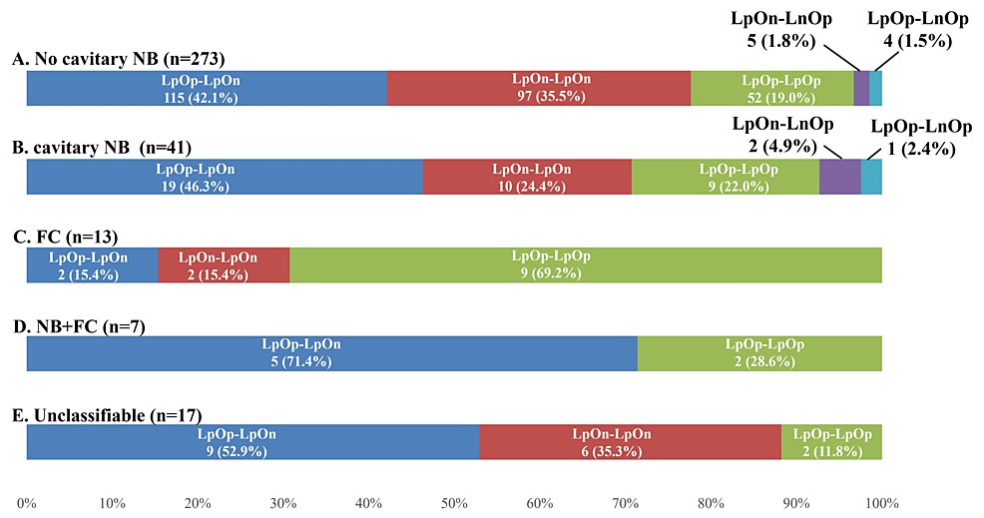
Sputum culture combinations for each radiological pattern. (A) Sputum culture combination for patients with no cavitary NB, (B) cavitary NB, (C) FC, (D) NB+FC, and (E) unclassifiable. "Lp" indicates a positive result on liquid culture, whereas "Ln" indicates a negative result. Similarly, "Op" indicates a positive result on Ogawa culture, whereas "On" indicates a negative result. LpOp-LpOp: positive on liquid culture and Ogawa culture twice; LpOn-LpOn: positive on liquid culture twice, but not Ogawa culture; LpOp-LpOn: positive once on both liquid culture and Ogawa culture and once only on liquid culture; LpOp-LnOp: positive once on both liquid culture and Ogawa culture and once only on Ogawa culture; LpOn-LnOp: positive once on liquid culture and once on Ogawa culture; FC: fibrocavitary; NB: nodular bronchiectatic

The breakdown of the sputum culture combination for each radiological pattern is also presented (Figure 7). LpOp-LpOn was the most common sputum culture combination except for FC, and LpOp-LpOp was the most common for FC only.

Culture results classified by combining smear results and radiological patterns

**Figure 8:**
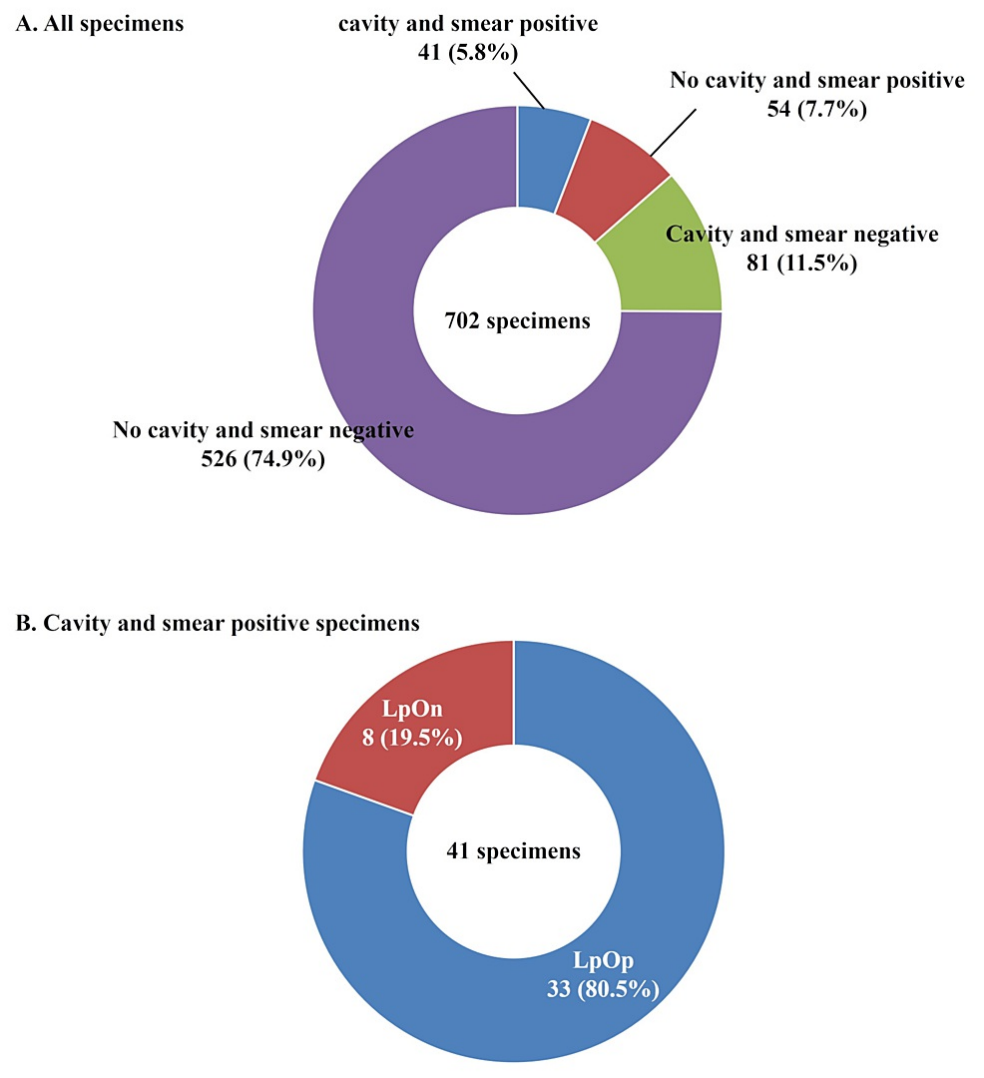
Sputum culture results for each smear and cavity. (A) Among all specimens, classification according to the presence of a cavity and smear results. (B) Among cavity and smear-positive specimens, sputum culture results in liquid and Ogawa media. "Lp" indicates a positive result on liquid culture, whereas "Ln" indicates a negative result. Similarly, "Op" indicates a positive result on Ogawa culture, whereas "On" indicates a negative result.

Among 122 specimens collected from patients with cavities (cavitary NB, FC, and NB+FC), 41 (33.6%) were smear-positive (Figure 8). Of the 580 specimens collected from patients without cavities, 54 (9.3%) were smear-positive. Of the 41 specimens that were smear-positive and had cavitary disease, 33 (80.5%) were LpOp, and eight (19.5%) were LpOn.

Differences in time to culture and in time from the first culture positivity to the second culture positivity

**Figure 9:**
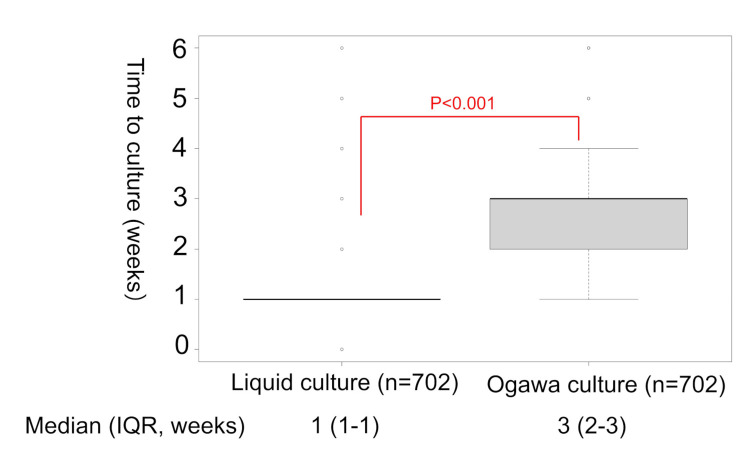
Time to culture in each medium.

Time to culture positivity was one (interquartile range (IQR), 1 to 1) week for LC and three (IQR, 2 to 3) weeks for OC (p-value <0.001) (Figure 9). The interval between the first culture positivity and the second culture positivity was a median of 56 days (IQR, 7-215 days).

## Discussion

LC was more sensitive and provided quicker culture results than OC. OC alone would have delayed diagnosis in 75.5% (265/351 patients). The positivity rate of OC varied depending on smear positivity or radiological pattern, and the consistent usefulness of LC was demonstrated. LC could detect MAC two weeks earlier than OC.

Some studies have shown that the detection sensitivity of mycobacterial species is higher when using both MGIT and solid medium, followed by using MGIT alone, and then using solid medium alone, and MGIT is faster than the Löwenstein-Jensen medium in terms of growth rate [9,10]. The MGIT culture system uses test tubes with a fluorescent indicator that becomes bright under ultraviolet light when oxygen is depleted due to the growth of mycobacteria and other organisms. This system is advantageous because it allows for easy detection of growth.

Although the use of LC alongside solid culture improves diagnostic yield, it also increases testing costs [7]. Whether there is a patient population for whom LC could be omitted based on smear results or radiographical patterns was analyzed. In terms of smear results, LC had a positivity rate of 98% (595/607 specimens) for smear-negative and 100% (95/95) for smear-positive specimens. In contrast, OC varied widely, with positivity rates of 40.2% (315/702) for smear-negative and 74.7% (71/95) for smear-positive specimens. Even for smear-positive specimens, OC alone could miss the MAC-LD diagnosis. In terms of radiological patterns, cavitary lesions on chest CT are said to suggest high bacterial loads and proliferation [11,12]. The positivity rate of OC was higher in FC>NB+FC>cavitary NB>non-cavitary NB>unclassifiable radiological patterns. Even in specimens collected from FC, NB+FC, and cavitary NB, which are considered to have relatively high bacterial abundance, 23.1% (6/26 specimens) to 50% (41/82) were negative with OC. Of the 41 specimens collected from radiological cavities and smear-positive cases, all cases cultured in LC were positive, whereas 33 specimens (80.5%) in OC were positive. In other words, it is difficult to predict in which cases OC alone will be sufficient based on radiological pattern and smear test results.

Lee et al. reported that there were cases that were positive on LC alone and positive on solid culture regardless of LC results [13]. They found that 11.3% (111/978 patients) of the cases were positive on LC alone, with a low smear-positive rate and percentage of cavities on radiography. They also reported that many of these patients, who were positive on LC alone, had mild symptoms and did not require induction of therapy. The degree of symptoms or whether treatment was subsequently initiated for MAC-LD was not examined. These findings suggest that the addition of LC to OC is preferable in cases with negative smears, with no cavities on radiography, or in which treatment should be initiated as soon as the MAC-LD diagnosis is made.

Urabe et al. reported the usefulness of three sputum specimens in 139 patients with the diagnosis of MAC-LD [14]. They used OC only and reported that only 16.5% (23/139 patients) of patients were diagnosed by three sputum examinations and 81.3% (113/139) required bronchoscopy. Although the number of cases requiring bronchoscopy for the MAC-LD diagnosis with OC alone is high, bronchoscopy may be avoided by using LC in combination with OC.

There are several limitations to the present study that should be noted, including its retrospective, single-centre design. There is potential for bias with regard to sputum sample quality, variation in the frequency of sputum tests between attending physicians, missing data, and radiological assessments.

## Conclusions

LC was more sensitive and provided quicker culture results than OC for early MAC-LD diagnosis. In cases with negative sputum smears and no cavities on radiography, combined culture using both LC and OC will be expected to facilitate early definitive diagnosis. In cases with positive sputum smears and cavities on radiography, OC may be sufficient in some cases, but should be aggressively combined with LC when a more rapid diagnosis is required.
